# Circadian clock—A promising scientific target in oral science

**DOI:** 10.3389/fphys.2022.1031519

**Published:** 2022-11-16

**Authors:** Guangxia Feng, Jiajia Zhao, Jinfeng Peng, Beibei Luo, Jiaqi Zhang, Lili Chen, Zhi Xu

**Affiliations:** ^1^ Department of Stomatology, Union Hospital, Tongji Medical College, Huazhong University of Science and Technology, Wuhan, China; ^2^ School of Stomatology, Tongji Medical College, Huazhong University of Science and Technology, Wuhan, China; ^3^ Hubei Province Key Laboratory of Oral and Maxillofacial Development and Regeneration, Wuhan, China

**Keywords:** circadian rhythm, circadian clock, oral diseases, oral and maxillofacial development, oral cancer

## Abstract

The oral and maxillofacial organs play vital roles in chewing, maintaining facial beauty, and speaking. Almost all physiological processes display circadian rhythms that are driven by the circadian clock, allowing organisms to adapt to the changing environment. In recent years, increasing evidence has shown that the circadian clock system participates in oral and maxillofacial physiological and pathological processes, such as jaw and tooth development, salivary gland function, craniofacial malformations, oral carcinoma and other diseases. However, the roles of the circadian clock in oral science have not yet been comprehensively reviewed. Therefore, This paper provides a systematic and integrated perspective on the function of the circadian clock in the fields of oral science, reviews recent advances in terms of the circadian clock in oral and maxillofacial development and disease, dialectically analyzes the importance of the circadian clock system and circadian rhythm to the activities of oral and maxillofacial tissues, and focuses on analyzing the mechanism of the circadian clock in the maintenance of oral health, affecting the common diseases of the oral and maxillofacial region and the process of oral-related systemic diseases, sums up the chronotherapy and preventive measures for oral-related diseases based on changes in tissue activity circadian rhythms, meanwhile, comes up with a new viewpoint to promote oral health and human health.

## 1 Introduction

Human activities follow a 24-h rhythm, and the endogenous clock system drives physiological and behavioral rhythms, enabling organisms to track daytime and adapt to predictable and recurrent daily changes (Panda, 2016). Recently, a series of studies have shown that the circadian rhythm/clock affects oral and maxillofacial growth and development, including the coordination of the maxilla and mandible, remodeling of the alveolar bone, tooth development, oral epithelium homeostasis, salivary gland growth, and saliva production. Rhythm disturbance and dysfunction of the circadian clock might result in diverse oral-maxillofacial pathological conditions, such as skeletal mandibular hypoplasia (SMH), Sjögren’s syndrome, and oral carcinoma. Reasonable adjustment of the daily routine following the circadian rhythm will have a positive impact on the prevention and prognosis of oral-maxillofacial diseases (Papagerakis et al., 2014a; De Assis and Oster, 2021). This paper reviewed research progress in terms of the circadian rhythm/clock in the oral science fields, including oral-maxillofacial development and related diseases.

## 2 Regulatory system of the circadian clock

The circadian clock exerts a pivotal regulatory effect in controlling a plethora of activities and functions in the organism, including organism development and diseases in mammals (Liu et al., 1997; Mendoza-Viveros et al., 2017). The internal circadian clock system controls the circadian rhythm, including the central circadian clock and the peripheral circadian clock, in organisms. The central circadian clock is located in the suprachiasmatic nucleus (SCN), which is regarded as the pacemaker of the circadian rhythm. The circadian clock system has a hierarchical architecture, in which the hypothalamic SCN functions as a light-responsive central clock generating neural and hormonal signals for peripheral clocks (Koike et al., 2012; Hastings et al., 2018). Ablation of the SCN causes behavioral rhythms, endocrine activity, and body temperature to become arrhythmic (Earnest et al., 1999).

Peripheral molecular clocks generate a circadian rhythm that relies on autonomous transcriptional and translational feedback loops in a series of clock genes. A heterodimeric protein complex including “circadian locomotor output cycles kaput” (CLOCK) and “brain and muscle ARNT-like 1” (BMAL1) drives transcription through E-box elements to activate downstream transcriptional genes, including Period1(Per1), Period2(Per2), Period3(Per3), Cryptochrome1(Cry1), and Cryptochrome2(Cry2). In addition, “reverse orientation-erb” (Rer-erb) and “RAR-related orphan receptor” (Ror) participate in the rhythmic transcriptional activity of the molecular oscillator. Chondrocytes 1 (DEC1) and DEC2 are also regulators of the mammalian molecular clock, which repress the heterodimeric complex ClOCK/BMAL1-induced Per1 activation through direct protein‒protein interactions with Bmal1 and competition for E-box elements. Two classical branches participate in the peripheral circadian clock feedback loop. The main loop is the Clock/Bmal1-PERs/CRYs loop. CLOCK/BMAL1 combines with E-box elements to activate downstream transcriptional genes, including mPers and mCry. When PER and CRY proteins accumulate excessively, the CLOCK/BMAL1 complex is then inhibited from an activating state into an inhibitory state, combined with inhibiting the activity of downstream transcriptional genes. The supplementary regulators ROR/REV-ERB act on RORE elements, which also regulate the circadian clock feedback loop (Honma et al., 2002; Cho et al., 2012; Partch et al., 2014; Takahashi, 2017). The central and peripheral circadian clocks promote each other to ensure the regularity of biological activity and physiological states.

## 3 Circadian rhythm/clock genes regulate maxillofacial embryonic development

Oral and maxillofacial development is a delicate and complicated process that is not only regulated by the interaction of multiple molecules and environmental factors but also requires accurate control in space and time from a fertilized ovum to an adult (Xu et al., 2016). Precise regulation in the external mesenchyme controls the position, shape, and size of the oral and maxillofacial organs during embryonic development.

Most bone and connective tissues in the craniofacial complex and branchial arch structure are derived from the migration and differentiation of cranial neural crest cells (CNCCs) (Chai et al., 2000). The reciprocity and interconnections lead the various craniofacial epithelia to differentiate into complex tissues, such as Meckel’s cartilage, the mandible, teeth, the cementum, and periodontal ligaments (Yuan and Chai, 2019). It has been demonstrated that clock genes can be detected in neural spine cells in the early embryonic period and that clock genes are essential for embryonic and condylar development (Koussoulakou et al., 2009).

**FIGURE 1:**
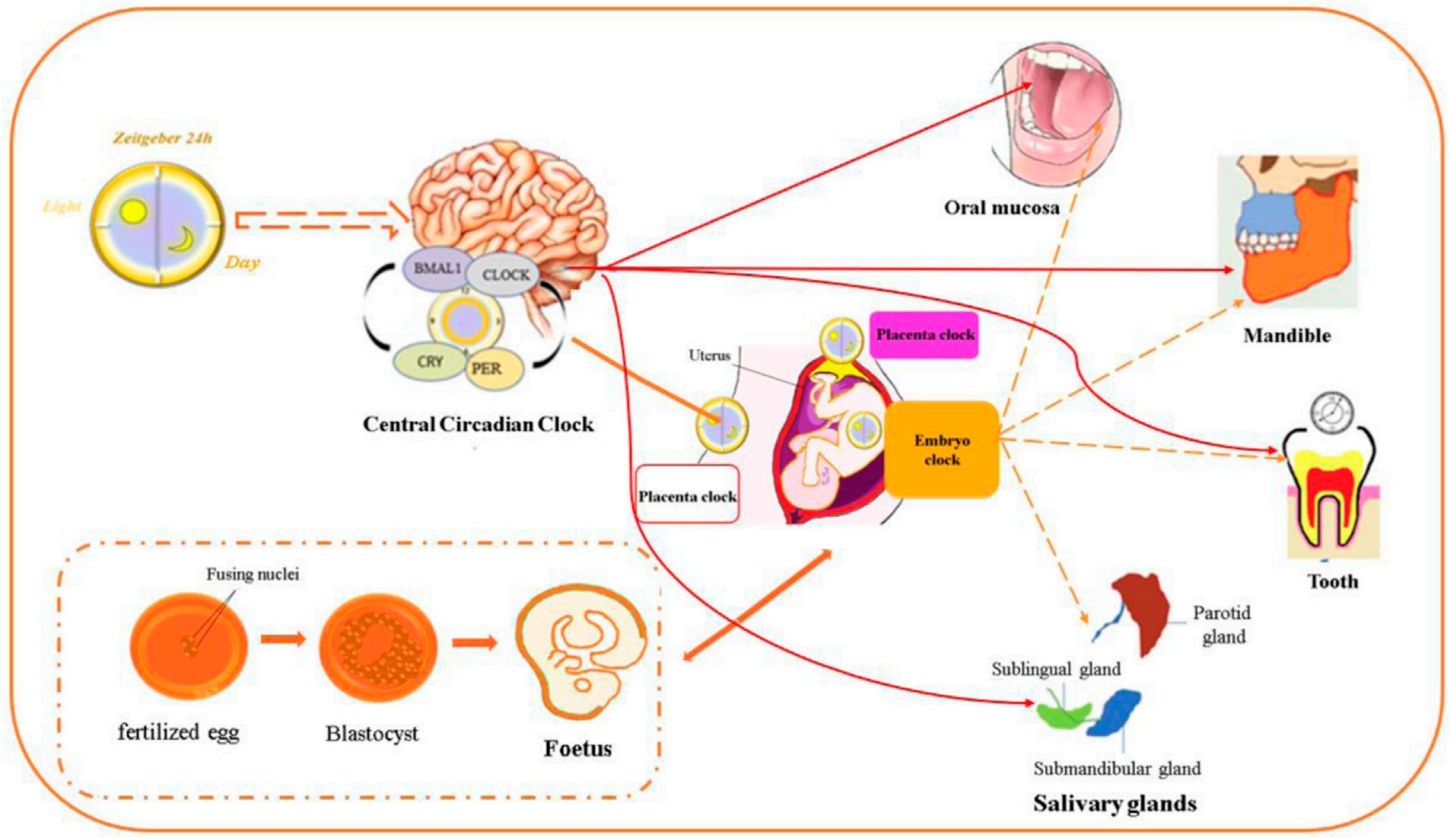
Circadian clock genes are expressed in the oral and maxillofacial region. The circadian clock regulates development in different stages of the embryonic period and postnatal stage. Circadian clock gene expression was detected in the maxillomandibular region, teeth, salivary glands, oral epithelium and periodontal tissue, which comprise the oral and maxillofacial regions.

The clock genes Bmal1, Clock, Pers, and Crys were detected in the oviduct and oocytes of fetuses and newborns. The downregulation of Bmal1 disrupts early embryonic development (Rivkees, 2003; Dekens and Whitmore, 2008; Xu et al., 2016; Challamel and Franco, 2018). Bmal1 and Clock, Per2, and Cry1 could be detected from the 10th day of pregnancy, and clock genes oscillate in the brain and organs of rat fetuses in a 24-h oscillation at 18 days of gestational age (Amano et al., 2009). Interestingly, the expression levels of Bmal1 and Per2 in the SCN and adrenal glands are higher than those in other tissues, indicating that the fetal primate SCN and adrenal glands are under different maternal rhythm control (Ibuka et al., 1977). It was revealed that the SCN is controlled by maternal melatonin, but the adrenal glands are regulated by undiscovered maternal signals. Changing maternal environmental factors could lead to abnormal signal transduction and induce craniofacial deformities. A substantial number of studies have shown that circadian clock genes can be detected at different levels and at various stages of growth and development in oral and maxillofacial complexes (Papagerakis et al., 2014a; De Assis and Oster, 2021). It is believed that many factors can change the circadian rhythm in the development of the oral and maxillofacial organs. The distal-less homeobox (Dlx), TGFβ, FGF23 and BMP2 signaling pathways are important for the development of CNCC-derived craniofacial tissues. These signaling pathways also present circadian expression patterns and interact with the molecular circadian oscillator in craniofacial differentiation (Kanzler et al., 2000; Chai et al., 2003; Choi et al., 2010; Carpinelli et al., 2017; Sloin et al., 2018). The craniofacial development-related genes also show rhythmicity in human mesenchymal stem cells during gastrulation, mesoderm formation, neural crest development, and epithelial-mesenchymal transition (Weger et al., 2017). These studies directly or indirectly proved that the circadian clock participates in the development of oral and maxillofacial tissues (Figure 1).

### 3.1 Roles of the circadian clock in dental development

Dental development is divided into two major stages: crown formation and root development. Teeth consist of enamel, dentin, cementum, and dental pulp (Dean, 2000). Tooth development stems from a sophisticated pattern of various signaling molecules and begins in the embryonic period controlled by reciprocal interactions of epithelial-mesenchymal tissues, which guides tooth development in the embryonic phases and generates dentin surface morphology when enamel is deposited (Chai et al., 2000; Yuan and Chai, 2019). During dental development, morphological changes during the differentiation of ameloblasts and odontoblasts are both correlated with periodic incremental lines regulated by a circadian rhythm (Zheng et al., 2013). Activation and maintenance of these periodic incremental lines recorded in mineralized dental tissues could be applied to explore the potential mechanism of dental development controlled by clock genes. There is a wealth of evidence demonstrating that the clock genes Bmal1, Clock, Per1, Per2, and Cry1 can be detected in different periods during tooth development and oscillate at regular 24 h intervals (Simmer et al., 2010).

These genes were detected in the bell stage for the first time and showed similar patterns of expression but exhibited different levels within ameloblasts and odontoblasts (Zheng et al., 2011). Recent studies have highlighted that Bmal1 and Cry1 are localized in the nucleus in ameloblasts and odontoblasts. Oscillation of Bmal1 and Per2 showed an antiphase in the synchronization of murine ameloblasts. The currently available evidence supports that rhythm-disturbed mice have significantly shorter incisor lengths than wild-type mice (Lacruz et al., 2012). Disruption of circadian rhythm in pregnant mice could result in enamel developmental retardation in their babies, enamel matrix secretion and amelogenin downregulated expression. It should be highlighted that melatonin receptors were discovered in tooth enamel, in pregnant mice, the melatonin receptor antagonist 4P-PDOT caused melatonin receptor and amelogenin (Amelx) deficiency in baby enamel tissue, and the physiological role of melatonin in tooth development may involve the modulation of mitochondrial function (Tao et al., 2016). The above studies indicate that circadian rhythm could affect normal enamel development at both pre- and postnatal stages *via* melatonin receptors. Circadian clock and ameloblast differentiation-related markers have potential temporal and spatial relationships. Clock genes are involved in amelogenin expression, enamel matrix secretion, cell junctions and arrangement, and mineralization during ameloblast differentiation, affecting the amelogenesis process (Athanassiou-Papaefthymiou et al., 2011). Studies indicated that secretory and maturation ameloblast markers amelogenin (Amelx), Ambn, Amtn, and Odam oscillated in a circadian pattern. Amelx and kallikrein-related peptidase 4 (Klk4) are downstream target genes of the circadian clock and show 24 h oscillatory expression patterns controlled by Bmal1 (Zheng et al., 2013). Mutations in KLK4 could cause nonsyndromic enamel malformations in humans and mice (Lu et al., 2008). The expression of AMELX and PPARγ was decreased in Per2 knockdown ameloblast lineage cells, and overexpression of PPARγ partially reversed this phenomenon, Per2 regulates ameloblast differentiation mainly *via* the PPARγ/AKT1/β-catenin signaling axis (Huang et al., 2021). Runx2 is involved in the whole process of tooth development, and Runx2 knockout mice are a representative congenital tooth agenesis model (Chen et al., 2020). Current studies support that Runx2 affects enamel formation and tooth development in a circadian clock-dependent manner. Runx2-mediated WNT and IGF signaling is critical for sustainable growth of the incisors, moreover, Runx2 activates the WNT inhibitor to regulate tooth root development in mice (Zheng et al., 2022). These studies identified the potential correlation between the circadian clock system and developmental controls of ameloblast differentiation. However, in a new study reported that the molars dentin underlie the ultradian rhythms with around 8-h periodicity in mouse, and upon ablation of SCN, the ultradian locomotor activity oscillations persisted in rodents under constant darkness or genetic disruption of the circadian clocks (Ibuka et al., 1977; Earnest et al., 1999; Zheng et al., 2014). These results revealed that the circadian clock does not seem to be involved in teeth development, which providided another understanding into the relationship between circadian rhythm and teeth growth.

**FIGURE 2:**
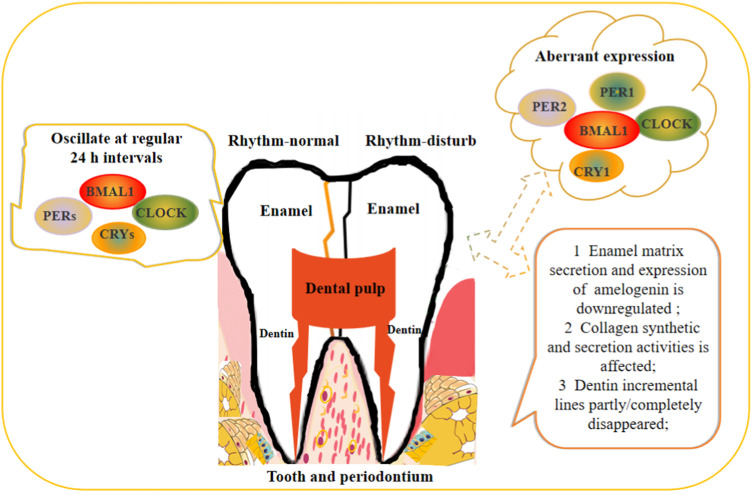
Schematic illustration of circadian rhythm regulates the teeth development.

In odontoblasts, collagen synthetic and secretion activities showed a circadian rhythm, and collagen secreted during the day is twice the amount secreted at night, which is likely to control the circadian incremental lines of dentin (Papakyrikos et al., 2020; Zheng et al., 2014) The clock proteins BMAL1, CLOCK, CRY1, PER1 and REV-ERBs are strongly expressed in odontoblasts, and the expression patterns are different in the crown and root dentin; the root is obviously strong (Zheng et al., 2011; Fu et al., 2022). Bmal1 promotes cementoblast differentiation and cementum mineralization *via* Wnt/β-catenin signaling (Yuan et al., 2022). Data obtained from *in vivo* studies of clock gene Bmal1 did not participate in the dentinogenesis process because the incremental lines persisted in both Bmal1 KO and wild-type mice housed in continuous darkness (Kondratov et al., 2006). Whether other clock genes have an impact on the dentin formation process is unknown. SCN is related to the incremental growth of dentin. In the case of bilaterally lesioned SCN, the dentin incremental lines completely disappeared while partially lesioned SCN only temporarily disturbed the dentin incremental line. This result suggested that the SCN affects the generation of the circadian dentin increment in rats (Ohtsuka-Isoya et al., 2001). Satomura K reported that ameloblasts, odontoblasts and dental follicle cells in third molars all express melatonin receptors, and melatonin could induce the proliferation of rat dental papilla cells *in vitro* and finally affect the formation of dentin (Tao et al., 2016). Odontoblasts are the target cells of various hormones, and these hormones are regulated by clock genes, indicating that odontogenesis is regulated by the circadian clock from the perspective of hormone levels (Zheng et al., 2014). In the three different embryonic phases of murine dental pulp cells, only Clock, Pers, and Bmal1 at low but variable levels were detected (Xu et al., 2022). Rev-erbαshowed oscillated expression in a 24 h cycle in cementoblast cell line OCCM-30, which suggested the circadian rhythms exist in cementoblasts. REV-ERBs negatively regulate cementoblasts mineralization through the inhibition of OSX, OCN, BSP, and ALP expression in cementogenesis (Fu et al., 2022). This study provided a potential target regarding periodontal and cementum regeneration. Dental pulp-derived stem cells show rhythmic oscillations of Bmal1, Rer-erbα, and Per2 under mechanical stretching (Tao et al., 2016). Dysfunction of the circadian clock affects dentin apposition and mineralization, the effects of BMAL1 on dentinogenic differentiation of DPSCs *via* PI3K/Akt/mTOR pathway (Xu et al., 2022), but the additional specific mechanisms underlying differential expression of circadian clock genes on tooth development needs to be further studied (Figure 2).

### 3.2 Craniofacial bone development and malformation regulated by the circadian clock

The maxilla and mandible together form the lower third of the facial skeleton, which performs important functions to maintain mastication, speech and aesthetics in daily life. The jaw is mainly deposited in the form of intramembranous ossification and endochondral (intracartilaginous) ossification (Ye et al., 2022). The length and width of the maxilla and mandible develop in the form of intramembranous ossification. The height and part of the length of the mandible depend on the endochondral ossification of the condylar cartilage. Circadian transcriptional components have been shown to coordinate oscillatory behavior in murine calvarial bone, and the related bone deposition and development are closely regulated by clock genes (Zvonic et al., 2007), (Sancar et al., 2010). Researchers used transgenic mice with a human osteocalcin promoter-related luciferase reporter gene to test the intensity of osteogenic activity at multiple skeletal sites, and the maxillomandibular complex showed the most robust oscillatory pattern at a periodicity of 24 h. Clock gene oscillation shows similarities and different amplitudes during different periods of development; the crests and troughs are close to each other (Gafni et al., 2009). Studies have demonstrated a distinct circadian rhythm in the growth and remodeling of mandibular and condylar cartilage in rats and mice, and the circadian rhythm could modulate endochondral bone formation to promote mandibular development mainly *via* the MTR1/AMPKβ1/BMAL1 signaling axis (Yu et al., 2022). During the development of the mandible in mice, BMAL1/CLOCK expression levels gradually decrease with increasing age, while the expression levels of PER1/2, REV-ERBα, CRY1/2, and RORα show no obvious change at different developmental stages of mandibular condylar cartilage (MCC) (Song et al., 2018a). Some bone metabolism-related markers, such as FGF23, CTX-I, melatonin, parathyroid hormone (PTH), P1NP, and osteocalcin proteins, display diurnal rhythmicity in a cosinor way over a 24-h period, and SCN output signals can regulate bone metabolism (Guo et al., 2015). Due to the limitation of the literature, the roles of REV-ERBα, CRYs, and RORα in jaw development also need further study.

Dysfunction of the circadian clock could affect homeostasis between osteoclast-mediated bone resorption and osteoblast-regulated bone formation in the development of the jawbone, leading to dysplasia (Zhao et al., 2018). Clock genes in osteoblasts maintain bone homeostasis mainly by regulating 1,25(OH)2D3-RANKl expression (Yang et al., 2019). The expressions of clock genes in mice mandibular tissues at different time points (ZT0, ZT4, ZT8, ZT12, ZT16, ZT20, and ZT24) were measured, compared with LD12:12 group, the expression rhythmic pattern characterized of clock genes Bmal1, Clock, Per1, Per2, Cry1, and Cry2 becomes out of arrhythmia in Jet Lag group (Zhao et al., 2018). Dysfunction of the circadian rhythm could slow down the rate of skeletal growth and development and is often accompanied by mandibulofacial dysostosis in adolescents. Zhao et al., (2018) confirmed that the circadian rhythm of the mandible was reset in patients with skeletal mandibular hypoplasia. The clock genes Bmal1, Clock, Per1, and Cry2 and bone-related markers were significantly decreased, and the reduction in Bmal1 was the most remarkable. Bmal1 downregulation caused by circadian disruption principally directly inhibits the transcription of OPG and indirectly increases Mmp3 expression through P65 phosphorylation, finally suppressing osteogenesis and promoting osteoclasis, resulting in bone loss (Zhou et al., 2018). Bmal1 knockout mice were accompanied by mandibular deformity. Yu et al. proved that BMAL1 deficiency inhibits endochondral ossification and chondrogenesis in a mandibular condyle by decreasing the differentiation of chondrocytes, BMAL1 deletion could induce thinning of the condylar cartilage proliferation layer and hypertrophy layer, decreasing chondrocyte proliferation and collagen deposition (Yu et al., 2020), and Bmal1 deletion could impair the function of chondrocytes by disrupting the HIF1α-VEGF signaling pathway (Ma et al., 2019). BMAL1 controls the downstream cascades of hedgehog signaling by directly binding to the Ptch1 and Ihh promoters, regulating targets of hedgehog signaling in craniofacial deformities (Yu et al., 2020). These studies indicated that BMAL1 could be a new biological marker for the diagnosis of jaw deformities and a new target for the prevention and treatment of jaw deformities.

In osteoblast-specific Bmal1 KO mice, the low bone mineral density phenotype is correlated with decreased osteogenic differentiation and increased osteoclast resorption, which mainly occurs through increased BMP2 signaling to promote trabecular bone formation (Takarada et al., 2017; Qian et al., 2020; Samsa et al., 2016; Serin and Acar Tek, 2019). Bmal1 regulates BMP2 expression to induce osteoblast differentiation (Min et al., 2016). It should be highlighted that conditional knockout of Bmal1 in endothelial cells and hematopoietic cells strongly enhances the cellular response to microvascular injury, which suggests that the circadian clock could affect bone metabolism by regulating angiogenesis, but the related mechanism needs further clarification (Lidington et al., 2022). Bmal1-and Per2-mediated Regulation of the Osteogenic Differentiation and Proliferation of Mouse BMSCs by Modulating the Wnt/β-catenin Pathway (Zheng et al., 2022). Deletion of Crys/Pers genes could also result in a high bone volume phenotype in mice (Song et al., 2018a). Cry2 could influence osteoclast activity, and Cry2-deficient mice display increased bone volume with low osteoclast activity (Destici et al., 2013). Per2 affects osteoblast parameters by increasing the bone formation rate without leptin (Destici et al., 2013). REV-ERB agonism partially upregulates FABP4 to prevent osteoclastogenesis (Song et al., 2018b).

**FIGURE 3:**
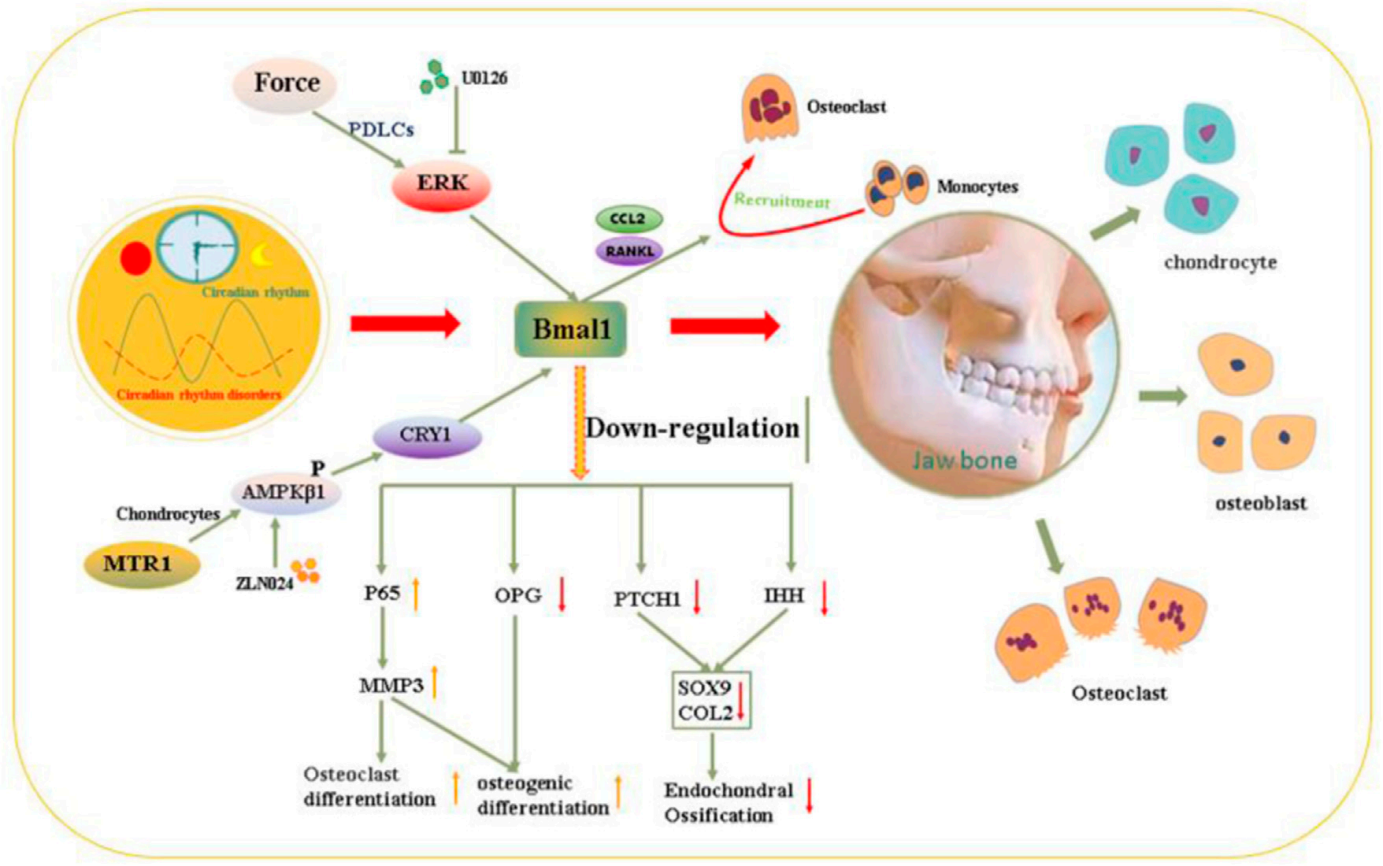
Molecular mechanism schematic illustration of the dysrhythmia affect osteogenesis and osteoclast process leading to maxillofacial developmental deformities.

Circadian rhythms play an indispensable role in the development of jaws, and rhythm disorders are one of the risk factors for jaw development deformities (Yu et al., 2020). Orthodontic treatment is currently the main measure for jaw dysplasia, and multiple factors could affect the sensitivity of periodontal ligament cells to mechanical force and cause alveolar bone reconstruction. BMAL1 in PDLCs is highly involved in sensing and delivering biomechanical signals in this process. Orthodontic force upregulates BMAL1 expression in periodontal tissues in a manner dependent on extracellular signal–regulated kinase ERK and activator protein 1 AP1 (Xie et al., 2022). Overexpression of BMAL1 could enhance the secretion of C-C motif chemokine 2 and RANKL in PDLCs, which subsequently promotes the recruitment of monocytes that differentiate into osteoclasts (Selenica et al., 2013). Localized injection of the ERK phosphorylation inhibitor U0 (Fu et al., 2016), or the BMAL1 inhibitor GSK4112 suppressed ERK/AP1/BMAL1 signaling, which dramatically reduced osteoclastic activity on the compression side of a rat orthodontic model. Bmal1 could be a useful therapeutic target for controlling pathologic bone remodeling activities (Xie et al., 2022). Of course, in the prevention and treatment of jaw dysplasia, compliance with the rhythm of tissue remodeling could improve the effect of surgical correction while reducing adverse reactions. These studies, to some extent, provide an improved understanding of the versatile circadian clock system in controlling jawbone development (Figure 3).

### 3.3 The circadian clock participates in periodontal physiology and pathology

Periodontal tissue consists of the alveolar bone, root cementum, gingiva, and periodontal ligament. Periodontal remodeling is a necessary condition for tooth movement during orthodontic treatment and for maintaining periodontal health. Studies have shown that the circadian clock genes BMAL1, Crys, PERs, and DEC1/2 show rhythmic expression in human and mouse junctional epithelium, gingival fibroblasts, periodontal ligaments, and human mesenchymal stem cells (Hilbert et al., 2019a). The proliferation of alveolar periosteum and maxillary first molar cementum cells in mice has a circadian rhythm, while the expression of bone and periodontal tissue development and remodeling-related genes OPG, RANKL, RUNX2, OCN, OPN, COL1A1, POSTN, and IL1β have circadian rhythm, all of which share a correlation with the fluctuation of ARNTL expression (Tonna et al., 1987; Roestamadji et al., 2019). For example, the OPG/RANKL system is affected by the ARNTL-related circadian rhythm in periodontal ligament fibroblasts, and IL1β and TNF-α in human saliva, as markers of inflammation, are regulated by the circadian clock (Sehirli et al., 2021; Vansant et al., 2018). Ki67 expression in periodontal ligament fibroblasts is controlled by the circadian rhythm, and dysregulation might lead to excessive proliferation and oral tumor development (Li et al., 2018). The circadian rhythmicity of fibroblasts influences the actin dynamics of wound healing, after periodontal surgery, the inflammatory response and wound healing in the gingiva are regulated by the circadian clock (Hoyle et al., 2017). Obviously, periodontal tissue exhibits obvious rhythmic characteristics under physiological conditions.

Periodontitis is the sixth most common human chronic disease, affecting over 11.2% of the world’s population (Nwizu et al., 2000; Karaaslan and Dikilitaş, 2019). It is an immune and inflammatory response mediated by bacteria, dysbiosis between microbes and multifactorial host immune system effects (Socransky et al., 1998). Alveolar bone remodeling is coupled with the processes of bone formation by osteoblasts and bone resorption by osteoclasts to maintain dynamic self-balance (Hienz et al., 2015). Alveolar bone destruction is a key feature of periodontitis; alveolar bone regeneration is a difficult clinical challenge in the prevention and treatment of periodontal disease. Published articles have confirmed that periodontal tissue remodeling is rhythmic and that melatonin could benefit alveolar bone remodeling by decreasing the synthesis of RANKL and increasing the synthesis of OPG to promote osteoblast formation and decrease bone resorption (Deng et al., 2020). Glucocorticoids play a key role in the transmission of circadian timing from the SCN to peripheral osteoclasts, and the osteoclast peripheral clock might control the bone resorption circadian rhythm by regulating CTSK and NFATc1 expression in periodontitis (Fujihara et al., 2014). Cortisol mediates bone resorption, and the circadian rhythm of salivary cortisol is correlated with aggressive periodontal disease, which might cause dysbiosis of the periodontal environment (Mathew et al., 2019). Shift work, irregular sleep and diet, and high mental stress could all lead to rhythm disturbances and are strongly associated with periodontitis. Park JS et al. reported that workers with circadian rhythm disorders had higher rates of salivary and gingival crevicular fluid TNF-α levels, oral inflammation and periodontal inflammatory status (Roestamadji et al., 2019; Roestamadji et al., 2020). Higher TNF-α levels could cause a reduction in tooth attachment and alveolar bone resorption. TNF-α and other inflammatory factors are locally released, attach to plaques and promote osteoclast activation, particularly under inflammatory osteolysis conditions in periodontitis (Roestamadji et al., 2020). Inflammatory factors also react with stromal cells, the gingival epithelium and the periodontium to trigger bone resorption, and the activation of osteoclast precursor cells and their differentiation might be controlled by the RANKE/RANKEL/OPG system, which is regulated by the circadian rhythm (Han et al., 2018).

Data obtained from the above studies indicated that rhythm disturbance could lead to immune dysregulation and increased oxidative stress that exacerbates the development of periodontitis. The molecular nature of circadian rhythm regulation and maintenance is a circadian clock gene-dependent transcription-translation feedback loop (Gold and Kinrys, 2019). Compared to WT mice, the structurally unstable PDLs showed rapid periodontal destruction in Bmal1 knockout mice; in addition, the bone of the interalveolar septum was obviously deeper, and the bone mineral density was significantly lower (Koshi et al., 2020; Maria and Witt-Enderby, 2014). Circadian clock disruption could enhance SIRT1/NAD + dissociation, which promotes NF-κB transcriptional activity, and the active NF-κB pathway promotes cytokine expression during the inflammatory processes of gingivitis and periodontitis (Nakahata et al., 2009). The interaction between Bmal1 and NF-κB may be the key target mainly because Bmal1 could reduce bone resorption, and the active NF-κB pathway suppresses Bmal1 expression. The reduction in Bmal1 levels may trigger further production of TNF-α and other proinflammatory factors and exacerbate periodontal inflammation and bone loss in gingivitis and periodontitis (Sehirli et al., 2021).

Periodontal tissue, gingival crevicular fluid and saliva are rhythmic; for example, salivary cortisol expression was highest 30 min after waking up and lowest an hour before bedtime, which could facilitate the selection of the perfect time of detection markers during the clinical treatment process, thereby promoting more accurate clinical evaluation (Hilbert et al., 2019b; Pruessner et al., 1997). Salivary melatonin is closely related to clinical periodontal parameters; melatonin is inversely correlated with gingival inflammation parameters, which inhibit inflammation-induced alveolar bone loss and the expression of the proinflammatory factors TNF-α and IL-1β in periodontal tissue of periodontitis rats, and periodontal therapy can restore saliva melatonin (Konečná et al., 2021; Park and Tokura, 1999). Melatonin could help regulate the immune response and prevent periodontal tissue destruction. Studies from *in vitro* studies proved that melatonin combined with metronidazole reduced the expression of proinflammatory factors in the treatment of periodontitis; certainly, the drug combination is always better than the single drug (Pruessner et al., 1997; Carpentieri et al., 2017). The above results suggest that melatonin has the potential to become a risk indicator for evaluating the severity of periodontitis, and restoration of circadian rhythm is expected to become a new strategy for the prevention and treatment of periodontitis. It is not clear that in the course of clinical treatment, if the medication time can synergize with the expression of inflammatory factors for chronotherapy, the curative effect of periodontitis may be improved to a great extent.

It has long been confirmed that there is a relationship between periodontal pathogens and systemic diseases. In recent years, some scholars have proven that circadian rhythms are also closely correlated with oral-related systemic diseases, and periodontal pathogens act as a bridge between them (Preshaw et al., 2012; Kozaki et al., 2015). Xie et al. found that Porphyromonas gingivalis could downregulate the expression of BAML1 and lead to rhythm disorders and recruit DNA methyltransferase 1 by activating the TLR-NF-κB signaling axis to promote the degradation of BMAL1 after methylation, activate the NF-κB signaling pathway to formulate a positive feedback loop, and increase oxidative stress, thereby stimulating atherosclerosis (Xie et al., 2020). Drugs are used to restore body rhythm; in the meantime, antibacterial drugs are used to treat atherosclerosis, such as metronidazole, and the plaque area is significantly reduced. Recovery rhythm may become a crucial prevention and treatment strategy for promoting oral-related systemic diseases.

### 3.4 Functional regulation of salivary glands *via* the circadian clock

Saliva is an essential substance for regulating the oral microbiome and maintaining oral health, especially in oral disease prevention and oral infection control (Papagerakis et al., 2014a). Saliva mainly functions by forming salivary films to cover the oral cavity surface. It is secreted from the parotid, sublingual, submandibular and salivary glands and many other minor submucosal salivary glands in the oral cavity (Proctor, 2000). Three pairs of major salivary glands usually contribute more than 90% of the total amount of unstimulated saliva (Plangsangmas et al., 2020). The circadian rhythm of the salivary glands plays a key role in controlling nutrition intake and the defense system by affecting salivary flow and ionic composition (Wada et al., 2017). In recent studies, salivary flow rate and salivary secretion were shown to be correlated with circadian rhythms (Bellavía et al., 1992). The clock genes Bmal1, Per2, Cry1, Aqp5, and Ano1 mRNAs showed regular oscillatory patterns under both under both light/dark (LD) and complete-dark conditions (DD). The expression levels of Aqp5 and Ano1 peaked 6 h earlier under the DD condition than under the LD condition. Maintenance of the circadian rhythm of Aqp5 and Ano1 expression under the DD condition indicated that clock genes might regulate the rhythmic expression of Ano1 and Aqp5 and may control osmic gradients in SGs (Satou et al., 2019). Another study reported that clock gene mRNAs and clock proteins were found differentially expressed in the serous acini and duct cells of all major salivary glands (Zheng et al., 2012). A variety of hormones (melatonin and cortisol), growth factors, enzymes, immunoglobulins, and ions, including Na+, K+, HCO3−, and Cl−, showed robust rhythmicity; for example, cortisol excretion showed lower evening levels and higher morning levels in humans (Satou et al., 2019). Enzymatic activity and secretion rates of α-amylase present endogenous circadian rhythm in the parotid gland of young rats. The circadian rhythmicity of salivary IgA secretion was repressed by SCN lesions, indicating that the rhythmicity of the salivary glands might be regulated by SCN in mouse submandibular glands (Reinhardt et al., 2019). Saliva flow was altered in Per2 and Bmal1 knockout mice (Zheng et al., 2012). Aqp5 exerts a key effect on salivary fluid secretion and is regulated by the clock gene Bmal1 (Papagerakis et al., 2014a). Bmla1 overexpression could increase the expression levels of Aqp5. It indicated that salivary glands have a peripheral clock mechanism that functions both in normal light/dark conditions and in the absence of light (Zheng et al., 2012; Uchida et al., 2018). These findings might enrich our understanding of the control mechanisms between salivary and circadian clock. Some studies have proved that the melatonin receptor has high expression in the acinar epithelium of the embryonic submandibular gland, and melatonin may act as a bridge between the circadian clock system and the salivary gland, which would be a potential treatment target for salivary gland-related diseases (Zheng et al., 2012).

Normal salivary secretion and salivary flow rates are directly related to oral health. A reduced salivary flow rate is a key indicator of oral sicca syndrome, and salivary gland disorders are the main factor affecting the reduction in saliva flow rate. Many diseases affect the salivary secretion and secretory function of salivary glands, and chronic sialoadenitis and Sjögren’s syndrome (SS) cause oligoptyalism (Mese and Matsuo, 2007). SS with hyposalivation leads to nocturnal oral discomfort, dysphagia, dental caries, pararthria, and impaired taste. In primary Sjogren’s syndrome patients, core clock gene expression and salivary flow rates showed a decreasing trend in ductal cells and serous acinar cells of all primary salivary glands, which proved that the circadian clock affects saliva reduction and that disruption of the circadian clock contributes to the pathogenesis of Sjögren’s syndrome (Xiang et al., 2021). In summary, saliva biomarkers could be applied to the diagnosis and progression prediction of salivary gland diseases.

### 3.5 Oral malignant changes regulated by clock genes

Oral mucosa undergoes continual rapid self-renewal of its surface cells, which act as a protective barrier against a variety of physiological and pathological stimulus. Its functions are to protect deep tissues and organs against mechanical and chemical damage, including damage caused by microorganisms and metabolite toxins or the intake of carcinogens. Self-renewing stem cells have a pronounced circadian rhythm, and deletion of Bmal1 or Per1/Per2 might result in dormant stem cell progressive accumulation or depletion in the skin and mucous membrane (Papagerakis et al., 2014b). Circadian rhythm/clock gene expression could be detected in basal cells of the oral epithelium, including the palatal and junctional epithelia and the epithelial rests of Malassez surrounding the dental roots (Potten et al., 2002).

Oral carcinoma has an incidence of approximately 4.7–32.2/10,000 worldwide and often occurs in the lip, floor of the mouth, palate, tongue, buccal mucosa, and gingiva (Potten et al., 2002; Renzi et al., 2019). Oral carcinoma is a multifactorial disease, and DNA damage or mutation caused by long-term consumption of tobacco and alcohol, environmental factors, HPV infection and genetic polymorphisms are regarded as risk factors (Ragos et al., 2018; Warnakulasuriya and MacDonald, 1993). Data obtained from *in vitro* and *in vivo* studies demonstrated that clock genes, such as hClock, hBmal1, hPer1, hCry1, and hTim are expressed in the basal layer rich in stem cells of oral tissues and their peak rhythmic oscillation are closely related to different phases of the cell cycle (Bjarnason et al., 2001b). Furthermore, these clock genes directly or indirectly play an inhibitory role in the growth of oral tumors, mainly *via* the regulation of downstream target gene expression and participate in cell apoptosis, cell proliferation, cell cycle regulation and DNA damage repair (Bjarnason et al., 2001a). Tumorigenesis is mostly caused by dysfunction of the cell cycle in the oral epithelium, which mainly depends on the cyclin-CDK-cyclin-dependent kinase inhibitor regulatory network to maintain the cell functional cycle, and during mitosis, circadian clock disruption could affect mitotic activity because DNA is very fragile (Soták et al., 2014). The number of cells undergoing DNA synthesis showed a 24-h cyclical variation in the human oral epithelium and the critical cancer-related genes p53 and cyclinβ1 also followed the circadian rhythm (Johnson et al., 2011; Warnakulasuriya, 2009). P53 is a significant gene in terms of G1 markers and tumorigenesis, and its expression peaks at the same time as PER1. After knockdown of Per1 and Per2, P53 is downregulated in tumor cells (Zaid et al., 2018; Zhang et al., 2016). BMAL1 also showed the same peak as cyclin β1, a marker of M-phase (Tang et al., 2017). BMAL1 deletion could decrease P53 and PER expression and contribute to the dysregulation of DNA damage and tumor growth and uncontrolled cell proliferation (Hsu et al., 2012). These studies indicate the intimate relationship between the circadian clock and the cell cycle.

Compared with that in normal tongue epithelial tissues/human tongue keratinocytes, BMAL1 expression were decreased in tongue squamous cell carcinoma (TSCC) patients/SCC9, SCC25, CAL27 cancer cell line, meanwhile, the cancer cell line displayed defferent BMAL1 expression rhythmic pattern characterized by shorter cycle and weaker amplitude (Tang et al., 2017). Mechanistically, BMAL1 regulates the PI3K-AKT signaling axis to exert tumor suppressive properties. BMAL1 synergizes with paclitaxel in the optimal timeframe, which could increase the drug sensitivity of TSCC and improve the chronotherapeutic effect (Tang et al., 2019). PER1 is downregulated in oral squamous cell carcinoma (OSCC) (Gong et al., 2021). Aberrant expression of Per1 affects the pathological process of oral cancer by controlling the expression of matrix metalloproteinase-2 and the intracellular distribution of laminin receptors, Per1 deficiency *via* the AKT/mTOR pathway promotes OSCC progression (Li et al., 2016a). Per1 can also regulate downstream tumor-related genes Ki-67, p53, c-Myc, Bax, MDM2, MMP9, and Bcl-2 in HNSCC (head and neck squamous cell carcinoma) (Li et al., 2016b, Gery et al., 2006). PER1 and CLOCK are Potential circulating biomarkers for HNSCC (Hsu et al., 2014). Per1 plays an antiapoptotic role, and Per3 has proapoptotic effects during tumorigenesis. The expression level of Per1 is higher in cancer cells than in healthy gingival cells, while Per3 expression shows the opposite pattern (Sato et al., 2011; Chen et al., 2012). PER2 proteins are downregulated in oral cancer (Guo et al., 2020), and this decrease in PER2 affects the CDK/CKI cell cycle network and the levels of P53, moreover, it inhibits DNA adduct repair, potentiates the cytotoxicity of oxaliplatin and promotes cell apoptosis in oral squamous-cell carcinoma cells, therefore, periodic oxaliplatin administration in synergy with PER2-mediated PCNA transcription repression promotes chronochemotherapeutic efficacy of OSCC (Tang et al., 2019). Overexpression of Per2 suppresses OSCC progression *via* the PI3K/AKT/mTOR pathway to activate autophagy (Guo et al., 2020). PER2 in peripheral blood as a biomarker for drug application could improve the chemotherapeutic effect of OSCC (Bjarnason et al., 2001a).

**FIGURE 4:**
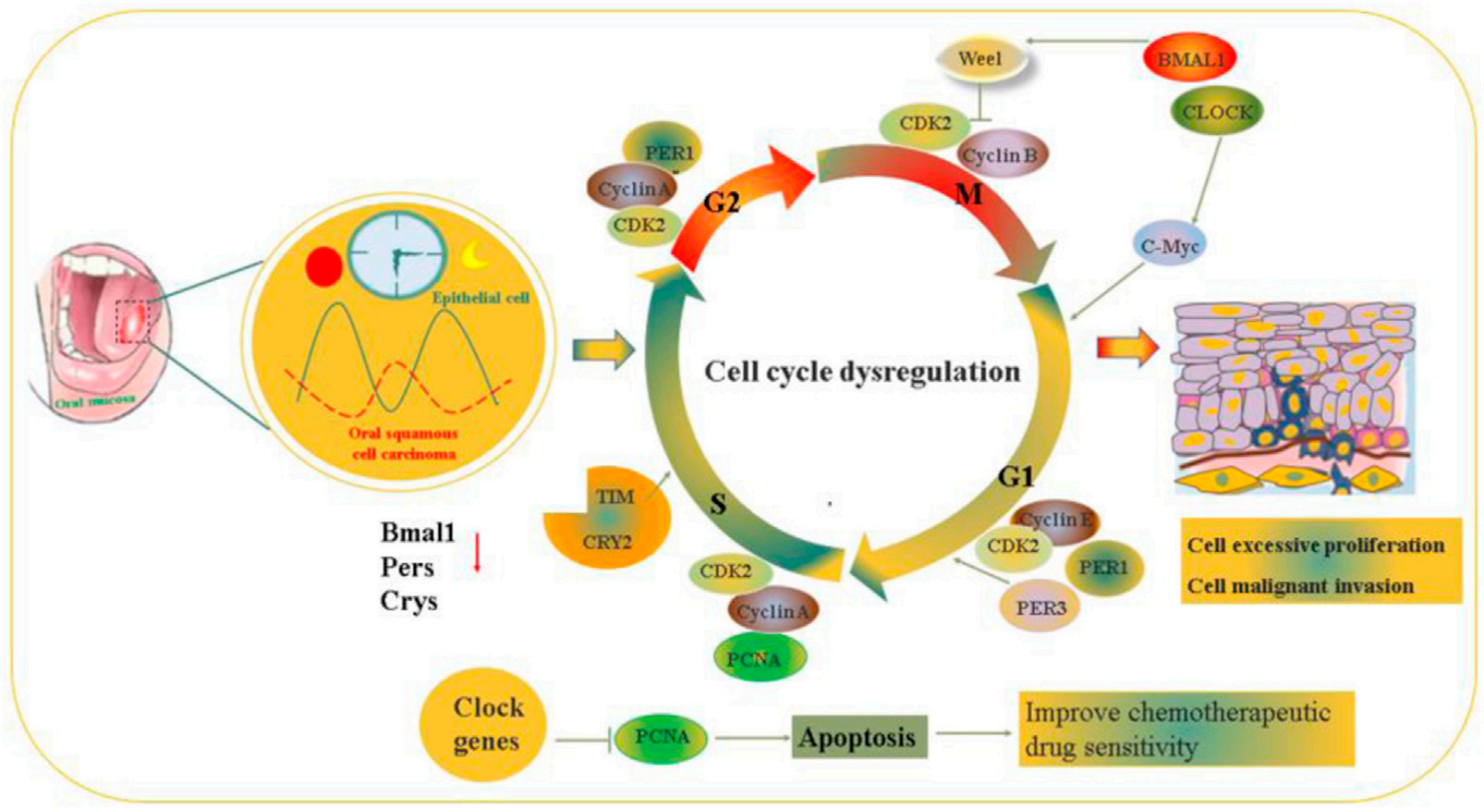
The molecular mechanism of circadian rhythm regulate the occurrence and development of the progressions on oral cancer *via* affecting cell proliferation and apoptosis.

In comparison to the pericarcinomatous tissue of head and neck squamous cell carcinoma (HNSCC) patients, clock genes Bmal1, Pers, and Crys, especially Cry2, showed a downward trend in cancer tissue (Tang et al., 2019). The volume and proliferation rate of oral tumors are related to circadian rhythm oscillations, which is similar to HNSCC (Okazaki et al., 2016). These studies indicated that chronochemotherapy could improve the efficacy and tolerance of oral cancer chemotherapy. Tang et al. have proved that BMAL1 in TSCC is a potential marker at the time of paclitaxel administration, and they showed that PER2 is a marker for tumor clinical stage and risk of metastasis as well as an ideal candidate for novel targets in the prevention and treatment of oral cancer (Tang et al., 2019) (Figure 4; Table 1).

**TABLE 1:** Expression of circadian clock in oral-craniofacial tissue.

Circadian clock genes	Associated tissues	Molecular functions	Animal models/Cell lines	Health	Disease	Related dysregulation/diseases
Clock	Teeth	Amelogenesis and dentinogenesis	Ameloblast cell line LS8	↑	↓	Enamel abnormality (Chen et al., 2020; Zheng et al., 2022)
		Dental pulp cells and dental pulp-derived cells development				
		Influences the rhythmic expression of ameloblast-specific genes and Runx2	Dental mesenchyme cell	↑	↓	
	Periodontal tissues	Maintains PDL stability				Not retrieved
		Stimulates SMAD3 promotor activity				
	Maxillomandibular Complex	Maintains maxillomandibular complex homeostasis	Mice			Not retrieved
		Regulates the maxillomandibular development	Mice			
	Oral mucosa	Maintain oral mucosa homeostasis				HNSCC (Li et al., 2016b; Hsu et al., 2014)
		Marker of good prognosis in HNSCC patients	Patients and healthy subjects	↑	↓	
Bmal1	Teeth	Bmal1 promotes cementoblast differentiation and cementum mineralization *via* Wnt/β-catenin signaling	Murine Cementoblast cell line OCCM-30	↑	↓	Defective amelogenesis (Lu et al., 2008), Dentinogenic differentiation disorder (Xu et al., 2022; Hsu et al., 2014)
		Ameloblasts and odontoblasts	Ameloblast cell OCCM-30	↑	↓	
		Contributes to the incremental lines of dentin and enamel	Mice	↑	↓	
		Affects dentinogenic differentiation of DPSCs *via* PI3K/Akt/mTOR pathway	DPSCs	↑	↓	
		Affects the ameloblast-specific genes Amelx and Klk4 expression				
	Periodontal tissues	Maintain gingiva and PDL stability and modulated by hypoxic condition	PDLs	↑	↓	Periodontitis (Tonna et al., 1987; Li et al., 2018; Roestamadji et al., 2019; Deng et al., 2020; Xie et al., 2020; Sehirli et al., 2021)
		Promotes SIRT1/NAD + dissociation and NF-κB transcriptional activity during the inflammatory processes	Mesenchymal stem cells			
		Correlated with interalveolar septum resorption	Mice PDLs	↑	↓	
	Maxillomandibular Complex	Force-induced expression of BMAL1 in PDLCs is closely involved alveolar bone remodeling	PDLCs	↑	↓	Skeletal mandibular hypoplasia (Zhou et al., 2018) Maxillofacial deformities (Yu et al., 2020; Ma et al., 2019; Min et al., 2016; Xie et al., 2022)
		Bmal Regulation of the Osteogenic Differentiation and Proliferation *Via* Wnt/β-catenin Pathway	Mouse BMSCs MC3T3-E1 cells Mandibular condylar chondrocyte	↑	↓	
		Regulates OPG, MMP3 expression through P65 signaling, resulting in osteogenesis	Rat BMSCs Human/Mice	↑	↓	
	Oral mucosa	Elevates the sensitivity of cancer cells to paclitaxel, as a chronotherapy timing biomarker in TSCC	Human tongue epithelial tissues/human tongue keratinocytes, SCC9, SCC25, and CAL27 cell lines	↑	↓	OSCC (Bjarnason et al., 2001a) TSCC (Guo et al., 2020)
		Inhibit tumorigenesis and increases paclitaxel sensitivity in TSCC		↑	↓	
	Salivary glands	Affects Aqp5 expression level and salivary fluid secretion/Alter saliva flow	Human, Mice	↑	↓	Sjogren’s syndrome (Zheng et al., 2012; Papagerakis et al., 2014a)
Per1/2/3	Teeth	Contributes to dental differentiation		↑	↓	Defective dentin mineralization (Tao et al., 2016)^,^ (Huang et al., 2021)
		Odontoblasts in crown and root	Mice			
		Ameloblasts and dental pulp cells	Mice			
		Regulates ameloblast differentiation *via* the PPARγ/AKT1/β-catenin signaling axis	ameloblast-lineage cells (ALCs)			
	Periodontal tissues	Periodontal ligament cells and gingival fibroblasts	Periodontal ligament cells			Not retrieved
		Maintain oral mucosa homeostasis				
	Salivary glands	Alters Saliva flow				Not retrieved
	Oral mucosa	Modulate cell proliferation, apoptosis and cell cycle progression Associated with carcinogenesis and later cancer stages; Perl *via* the AKT/mTOR pathway promotes OSCC progression; Regulation of the cycle in CDK/CKI cell cycle network in OSCC; Per2 suppresses oral squamous cell carcinoma progression by activating autophagy *via* the PDNAKT/mTOR pathway Associates with tumor development stage in HNSCC	Human tongue epithelial tissues/human tongue keratinocytes, SCC9, SCC25, and CAL27 cell lines	↑	↓	OSCC (Guo et al., 2020; Chen et al., 2012) HNSCC (Gery et al., 2006; Bjarnason et al., 2001a)
Cry1/2	Teeth	Ameloblasts, odontoblasts and dental pulp cells				None reported
		Regulated by hypoxic condition				
	Periodontal tissues	Fibroblasts of human gingiva and periodontal ligament				Not retrieved
	Oral mucosa	Modulate cell proliferation, apoptosis and cell cycle progression	Human tongue epithelial tissues/human tongue keratinocytes, SCC9, SCC25, and CAL27 cell lines	↑	↓	OSCC (Guo et al., 2020)
REV-ERBs	Periodontal tissues	Nuclei of cementoblasts around cellular cementum. REV-ERBs activation inhibits proliferation but promotes migration of cementoblast	Cementoblast cell line OCCM-30	↑	↓	None reported
	Teeth	Dental pulp-derived stem cells				

## 4 Perspective

Biorhythm disorders could lead to the development of malformations of oral and maxillofacial tissues, inflammation and tumor development by affecting cell proliferation, apoptosis, oxidative stress, *etc.*, and increase the incidence of oral diseases and oral-related systemic diseases (Qian et al., 2020; Konečná et al., 2021; Mese and Matsuo, 2007). With the deepening of circadian clock research, many methods can be applied to clinical prevention and treatment, such as small molecule targeted drugs or gene therapy, to improve rhythm disorders, effectively promote jaw development, relieve periodontal inflammation, and further improve the efficacy of oral cancer chemotherapy drugs and tolerance. Meanwhile, choosing the right opportunity to apply orthodontic force or administer chemotherapy drugs could effectively maintain the effect of bone remodeling, reducing complications, including root resorption and loosening caused by orthodontic force, and poor prognosis of radiotherapy and chemotherapy, laying the foundation for personalized chronotherapy. The circadian clock may be applied as a marker and intervention target for oral diseases and oral-related systemic diseases. Biochronotherapy is expected to become an effective clinical prevention and treatment measure for oral and maxillofacial-related diseases to promote general health and achieve personalized precision medicine. Of course, numerous current applications are restricted to animal-level studies, and much still remains to be done to promote the clinical translation of treatments.
